# Effects of transversus abdominis plane block versus quadratus lumborum block on postoperative analgesia: a meta-analysis of randomized controlled trials

**DOI:** 10.1186/s12871-020-01000-2

**Published:** 2020-05-04

**Authors:** Yanqing Wang, Xiaojia Wang, Kexian Zhang

**Affiliations:** 1 Department of Anesthesiology, Sichuan Cancer Hospital & Institute, Sichuan Cancer Center, School of Medicine, University of Electronic Science and Technology of China, No.55, Section 4, South Renmin Road, Chengdu, 610041 People’s Republic of China; 2 Department of Pain management, West China Hospital, Sichuan University, Chengdu, 610041 People’s Republic of China

**Keywords:** Transversus abdominis plane block, Quadratus lumborum block, Postoperative analgesia, Outcomes

## Abstract

**Background:**

Trunk block technique has been used in postoperative analgesia for patients undergoing surgery, specifically, transversus abdominis plane block (TAPB) and quadratus lumborum block (QLB) have been proved effective. The purpose of this meta-analysis is to evaluate the effects of TAPB and QLB in postoperative analgesia.

**Methods:**

Online databases, including MEDLINE, EMBASE, Cochrane Library (&Trail), Web of Science, CNKI, Wanfang and QVIP were applied to collect the randomized controlled trials (RCTs) from inception to Dec. 9th, 2019. Twenty-two studies were finally included containing 777 patients in the TAPB group and 783 cases in QLB group. RCTs comparing TAPB and QLB in postoperative analgesia were included in this meta-analysis. The indicators including total analgesia consumption postoperatively, operative time, duration of anesthesia, visual analogue scale (VAS) score at 24 h postoperatively, duration of postoperative analgesia, the number of patients requiring analgesia postoperatively and adverse reactions were analyzed.

**Results:**

our findings showed that morphine consumption (mg) (WMD = 3.893, 95%CI: 2.053 to 5.733, *P* < 0.001), fentanyl consumption (μg) (WMD = 23.815, 95%CI: 15.521 to 32.109, *P* < 0.001), VAS score at 24 h postoperatively (WMD = 0.459, 95%CI: 0.118 to 0.801, *P* = 0.008), the number of patients requiring analgesia postoperatively (WMD = 3.893, 95%CI: 2.053 to 5.733, *P* < 0.001), and the incidence of dizziness (WMD = 2.691, 95%CI: 1.653 to 4.382, *P* < 0.001) in TAPB group were higher than in QLB group.

**Conclusions:**

QLB is superior to TAPB in reducing morphine consumption, fentanyl consumption, VAS score at 24 h postoperatively, the number of patients requiring analgesia postoperatively, and the incidence of dizziness.

## Background

Postoperative pain, including acute postoperative pain and persistent chronic postoperative pain, remains a main clinical problem. Without timely and effective treatment, acute postoperative pain can turn into persistent chronic postoperative pain [1]. Previous studies showed that 10–50% of patients undergoing surgery suffered from postoperative pain lasting more than 1 month, and 2–10% of these patients continued to experience moderate to severe chronic pain. Furthermore, inadequate postoperative analgesia continues to occur despite advances in analgesia techniques [2, 3]. Inadequate management of postoperative pain can lead to serious consequences, such as poor immediate postoperative effect, prolonged stay and/or hospital readmission, poor patient satisfaction, increased burden on patients and health systems [3, 4]. Therefore, effective prevention and control of postoperative pain is of great significance.

Multimodal analgesia technique has been widely applied in postoperative analgesia [3, 5]. Truncal block, including transversus abdominis plane block (TAPB), quadratus lumborum block (QLB), rectus sheath block and hernia block, plays important roles in multimodal analgesia [6, 7]. TAPB involves injecting local anesthetic into the plane between the transverse abdominis and the internal oblique, it can block the sensory nerve supply to the anterior abdominal wall by deposition of local anesthetics and has shown promising in managing postoperative pain [8–10]. QLB, similar to TAPB, was first introduced as a different form of TAPB in 2007 [11]. It is also known as an interfascial plane block because it involves injecting local anesthetics into the thoracolumbar fascia which is different from TAPB. QLB can result in a widespread sensory suppression via a wide distribution of local anesthetics, and has been increasingly used for postoperative analgesia [11–14].

In recent years, many randomized controlled trials (RCTs) have been conducted to compare the effects of TAPB and QLB in postoperative analgesia [6, 15–18]. However, the results of outcomes of postoperative analgesia were inconsistent. In the current study, we aimed to compare the efficacy of TAPB versus QLB in postoperative analgesia based on RCT articles with a meta-analysis. The indicators for this meta-analysis included total analgesia consumption postoperatively, operative time, duration of anesthesia, visual analogue scale (VAS) score at 24 h postoperatively, duration of postoperative analgesia, the number of patients requiring analgesia postoperatively and adverse reactions.

## Methods

### Search strategy

The literatures were retrieved from MEDLINE, EMBASE, Cochrane Library (&Trail), Web of Science, CNKI, Wanfang and QVIP the deadline for searching documents was Dec. 9th, 2019. The index words for searching literatures as follows: ‘transversus abdominis’ OR ‘transversus abdominis plane block’ OR ‘transverse abdominis’ OR ‘transverse abdominis plane block’ OR ‘TAP’ OR ‘TAP block’ OR ‘TAPB’ AND ‘quadratus lumborum’ OR ‘quadratus lumborum block’ OR ‘quadrate lumborum’ OR ‘quadrate lumborum block’ OR ‘QL’ OR ‘QL block’ OR ‘QLB’.

### Inclusion and exclusion criteria

Inclusion criteria: (1) RCTs; (2) comparison of TAPB and QLB in postoperative analgesia; (3) English and Chinese literatures; (4) outcome indicators: total analgesia consumption postoperatively, operative time, duration of anesthesia, VAS score at 24 h postoperatively, duration of postoperative analgesia, the number of patients requiring analgesia postoperatively and adverse reactions.

Exclusion criteria: (1) reviews, meta-analyses, conference articles and letters; (2) animal experiments; (3) repetitive studies; (4) articles that cannot extract the valid data.

### Methodological quality appraisal

The studies were screened independently by two researchers Y Wang and X Wang. In the event of disagreements, a third party (K Zhang) would participate in the discussion. The modified Jadad scale (Table 1) was applied to evaluate the quality of literatures. The scale was divided into 7 points, 1–3 were defined as low quality, and 4–7 were defined as high quality.

**Table 1:** The modified Jaded Scale

Classification	Score	Description
Randomization		
Inappropriate	0	Semi-randomized or quasi-randomized trials
Unclear	1	Randomized trials without describing methods for generating random sequences
Appropriate	2	Random sequences produced by a computer or a random number table
Allocation concealment		
Inappropriate	0	Regular grouping
Unclear	1	Only use of a random number table or other random assignment scheme
Appropriate	2	A method for assigning sequences without prediction
Blinding		
Inappropriate	0	Use of double blindness without an appropriate method
Unclear	1	Only mention of double blindness
Appropriate	2	A description of the specific and appropriate method of double blindness
Withdrawals or dropouts		
No	0	No description of withdrawal or dropouts
Yes	1	A description of withdrawal or dropouts

### Statistical analysis

Heterogeneity test was conducted for each indicator and measured by statistics of I^2^, with I^2^ > 50% indicating significant heterogeneity. If I^2^ > 50%, a random effects model was used; if I^2^ < 50%, the fixed effects model was applied, and the heterogeneity was assessed. The software Stata 15.0 (Stata Corporation, College Station, TX, USA) was used for statistical analysis, effect index relative risk (RR) was used for enumeration data and weighted mean difference (WMD) for measurement data. *P* < 0.05 was considered statistically significant.

## Results

### Included studies

**Fig. 1:**
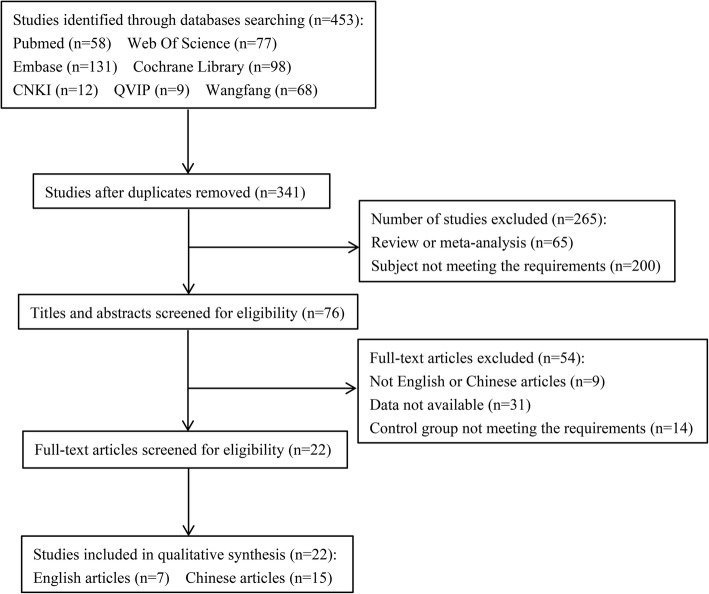
Flow chart of the review process

According to the search strategy, literature searches via the databases identified 453 articles. Following removing duplicates, screening titles or abstracts, and after assessing the full texts of relevant studies, 22 articles [6, 15–35] were finally included containing 777 patients in the TAPB group and 783 cases in QLB group (Table 2 and Fig. 1).

**Table 2:** Characteristics of studies included in meta-analysis

Author	Year	Country	Score	Treatment	TAPB_n (M/F)	TAPB_age^#^ (years)	QLB_n (M/F)	QLB_age^#^ (years)	Quality	Outcomes
Baytar	2019	Turkey	4	TAPB vs QLB	53 (11/42)	48.12 ± 12.42	54 (15/39)	46.42 ± 16.57	HQ	b f g
Yousef	2018	Egypt	5	TAPB vs QLB	30 (0/30)	50.70 ± 6.8	30 (0/30)	56.5 ± 6.97	HQ	a b c d e f
Kumar	2018	India	4	TAPB vs QLB	35 (15/20)	38.34 ± 11.59	35 (15/19)	39.20 ± 11.64	HQ	a b d f
Öksüz	2017	Turkey	3	TAPB vs QLB	25 (21/4)	3.02 ± 1.82	25 (21/4)	3.13 ± 0.20	LQ	e f
Blanco	2016	Arab	3	TAPB vs QLB	38 (0/38)	NA	38 (0/38)	NA	LQ	a
Verma	2019	India	6	TAPB vs QLB	30 (0/30)	28 ± 3	30 (0/30)	30 ± 3	HQ	b d f
Ipek	2019	Turkey	3	TAPB vs QLB	29 (19/10)	4.16 ± 2.55	35 (28/7)	3.89 ± 3.26	LQ	e f g
Shan	2019	China	3	TAPB vs QLB	30 (0/30)	30 ± 3	30 (0/30)	29 ± 6	LQ	c f
Deng	2019	China	6	TAPB vs QLB	34 (12/22)	53.5 ± 10.6	34 (14/20)	51.1 ± 13.8	HQ	b c f
Fu	2019	China	4	TAPB vs QLB	30 (NA)	71.8 ± 5.8	30 (NA)	72.2 ± 6.9	HQ	b f
Han	2017	China	4	TAPB vs QLB	38 (24/14)	27.8 ± 3.9	39 (20/19)	26.3 ± 3.2	HQ	b c f
He	2018	China	2	TAPB vs QLB	36 (20/16)	67.3 ± 2.3	36 (19/17)	67.7 ± 2.1	LQ	e f
Li G	2018	China	5	TAPB vs QLB	40 (0/40)	31 ± 4	40 (0/40)	30 ± 5	HQ	b c f
Li N	2019	China	3	TAPB vs QLB	30 (0/30)	42.10 ± 5.26	30 (0/30)	41.07 ± 4.75	LQ	b e f
Ma	2019	China	3	TAPB vs QLB	30 (17/13)	55. 2 ± 4. 4	30 (16/14)	53.1 ± 4.6	LQ	e
Ren	2018	China	3	TAPB vs QLB	82 (44/38)	45.7 ± 15.2	78 (40/38)	46.3 ± 15.1	LQ	b c
Xia	2018	China	4	TAPB vs QLB	30 (15/15)	48 ± 8	30 (12/18)	46 ± 11	HQ	f
Yang	2019	China	3	TAPB vs QLB	30 (0/30)	NA	30 (0/30)	NA	LQ	a b
Yang	2019	China	5	TAPB vs QLB	30 (0/30)	38.5 ± 14.8	30 (0/30)	43.9 ± 15.04	HQ	b c e f
Ye	2019	China	4	TAPB vs QLB	28 (12/16)	48.9 ± 2.1	30 (14/16)	50.3 ± 2.8	HQ	c f
Zhu	2019	China	3	TAPB vs QLB	39 (20/19)	68.8 ± 3.4	39 (18/21)	69.1 ± 3.2	LQ	e f
Zhu	2018	China	3	TAPB vs QLB	30 (0/30)	52 ± 6	30 (0/30)	51 ± 7	LQ	b e f

^#^: mean ± standard deviation

*TAPB* transversus abdominis plane block, *QLB* quadratus lumborum block, *VAS* visual analog scale, *HQ* high-quality, *LQ* low-quality, *NA* unavailable

a: morphine consumption; b: VAS score at 24 h postoperatively; c: fentanyl consumption; d: duration of postoperative analgesia; e: the number of patients requiring analgesia postoperatively; f: operative time; g: duration of anesthesia

### Overall meta-analysis

As shown in Table 3, our findings showed that morphine consumption (mg) (WMD = 3.893, 95%CI: 2.053 to 5.733, *P* < 0.001), fentanyl consumption (μg) (WMD = 23.815, 95%CI: 15.521 to 32.109, *P* < 0.001), VAS score at 24 h postoperatively (WMD = 0.459, 95%CI: 0.118 to 0.801, *P* = 0.008), the number of patients requiring analgesia postoperatively (WMD = 3.893, 95%CI: 2.053 to 5.733, *P* < 0.001), and the incidence of dizziness (RR = 2.691, 95%CI: 1.653 to 4.382, *P* < 0.001) in TAPB group were higher than in QLB group. No significant differences were observed between the two groups regarding the operative time (min) (*P* = 0.573), duration of anesthesia (min) (*P* = 0.733), duration of postoperative analgesia (h) (*P* = 0.258), and nausea and vomiting (*P* = 0.141).

**Table 3:** Overall results of the meta-analysis

Outcomes	WMD/RR (95%CI)	*P*	I^2^
Morphine consumption (mg)			
Overall	3.893 (2.053, 5.733)	< 0.001	72.7
Operation types			
Abdominal surgery	2.400 (1.825, 2.975)	< 0.001	NA
Pelvic surgery	4.731 (2.634, 6.829)	< 0.001	44.5
Quality			
High-quality	3.205 (1.283, 5.127)	0.001	76.2
Low-quality	6.443 (0.098, 12.788)	0.047	72.2
Fentanyl consumption (μg)			
Overall	23.815 (15.521, 32.109)	< 0.001	96.0
Operation types			
Abdominal surgery	14.077 (7.412, 20.742)	< 0.001	92.3
Pelvic surgery	34.808 (14.079, 55.537)	0.001	96.5
Quality			
High-quality	26.576 (13.594, 39.558)	< 0.001	96.9
Low-quality	16.264 (7.527, 25.000)	< 0.001	73.3
Operative time			
Overall	0.324 (−0.805, 1.454)	0.573	0.0
Duration of anesthesia (min)			
Overall	-2.139 (−14.423, 10.146)	0.733	80.8
VAS score at 24 h postoperatively			
Overall	0.459 (0.118, 0.801)	0.008	94.8
Operation types			
Abdominal surgery	0.224 (−0.033, 0.480)	0.088	80.1
Pelvic surgery	0.671 (0.103, 1.240)	0.021	95.4
Quality			
High-quality	0.576 (0.048, 1.104)	0.032	96.3
Low-quality	0.218 (−0.019, 0.455)	0.071	66.1
Duration of postoperative analgesia			
Overall	-21.882 (−59.774, 16.010)	0.258	100.0
Operation types			
Abdominal surgery	-3.400 (−4.038, −2.762)	< 0.001	NA
Pelvic surgery	−31.125 (−78.851, 16.600)	0.201	100.0
Number of patients requiring analgesia postoperatively			
Overall	2.618 (2.040, 3.361)	< 0.001	13.2
Adverse reactions			
Dizziness			
Overall	2.691 (1.653, 4.382)	< 0.001	0.0
Nausea and vomiting			
Overall	1.918 (0.805, 4.571)	0.141	50.9
Quality			
High-quality	4.100 (1.932, 8.699)	< 0.001	0.0
Low-quality	0.417 (0.054, 3.239)	0.403	70.9

*CI* confidence interval, *RR*, risk ratio, *WMD* weighted mean difference, *VAS* visual analog scal, *NA* unavailable

### Total analgesia consumption postoperatively

**Fig. 2:**
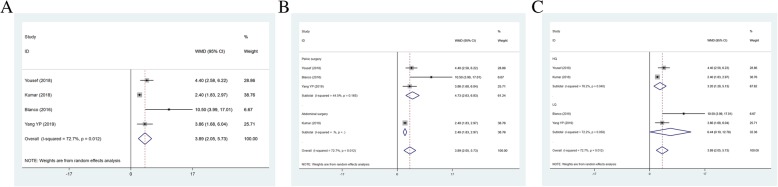
Forest plot for morphine consumption (**a**), operation types (**b**) and literature quality (**c**)

**Fig. 3:**
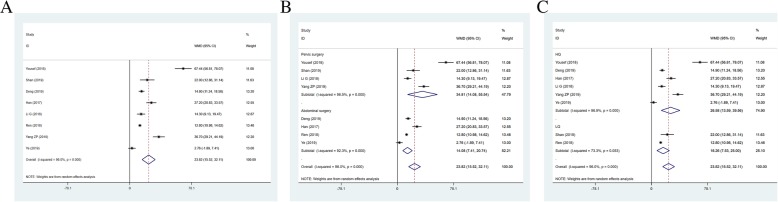
Forest plot for of fentanyl consumption (**a**), operation types (**b**) and literature quality (**c**)

Total analgesia consumption postoperatively (mg) as an outcome was reported containing 4 studies (*n* = 266) on morphine consumption (mg) and 8 articles (*n* = 623) on fentanyl consumption (μg). Patients in TAPB group consumed more morphine than QLB group (WMD = 3.893, 95%CI: 2.053 to 5.733; *P* < 0.001) (Table 3 and Fig. 2a). Heterogeneity among the included studies was statistically significant (I^2^ = 72.7%). Subgroup analysis was performed to identify sources of heterogeneity. According to operation types and literature quality, there were significant differences in abdominal surgery (WMD = 2.400, 95%CI: 1.825 to 2.975, *P* < 0.001), pelvic surgery (WMD: 4.731, 95%CI: 2.634 to 6.829, *P* < 0.001), high-quality (WMD = 3.205, 95%CI: 1.283 to 5.127, *P* = 0.001) and low-quality (WMD = 6.443, 95%CI: 0.098 to 12.788, *P* = 0.047) between the two groups (Fig. 2b and c). The fentanyl consumption in TAPB group was higher than that in QLB group (WMD = 23.815, 95%CI: 15.521 to 32.109, *P* < 0.001) (Table 3 and Fig. 3a). We also found statistical differences in abdominal surgery (WMD = 14.077, 95%CI: 7.412 to 20.742, *P* < 0.001), pelvic surgery (WMD: 34.808, 95%CI: 14.079 to 55.537, *P* < 0.001), high-quality (WMD = 26.576, 95%CI: 13.594 to 39.558, *P* < 0.001) and low-quality (WMD = 16.264, 95%CI: 7.527 to 25.000, *P* < 0.001) between the two groups (Fig. 3b and c).

### Operative time

Eighteen articles (*n* = 1204) on operative time (min) were included containing 597 patients in TAPB group and 607 patients in QLB group. The operative time in TAPB group was similar to QLB group, with no significant differences (WMD = 0.324, 95%CI: − 0.805 to 1.454, *P* = 0.573).

### Duration of anesthesia

The data of duration of anesthesia (min) as a clinical outcome was extracted from 2 articles including 171 cases. Duration of anesthesia in TAPB group was near to QLB group, with no significant differences (WMD = -2.139, 95%CI: − 14.423 to10.146, *P* = 0.733).

### VAS score at 24 h postoperatively

**Fig. 4:**
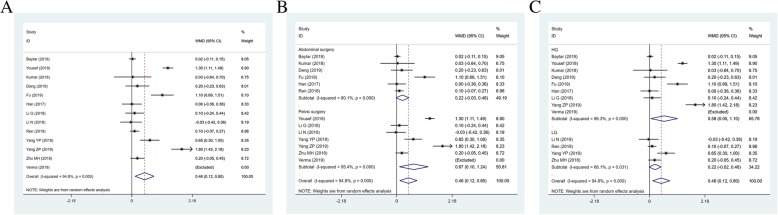
Forest plot for VAS score at 24 h postoperatively (**a**), operation types (**b**) and literature quality (**c**)

Thirteen studies, including 982 patients, reported VAS score at 24 h postoperatively for pain as an outcome (I^2^ = 94.8%). The VAS score at 24 h postoperatively in TAPB group was higher than that in QLB group (WMD = 0.459, 95% CI: 0.118 to 0.801; *P* = 0.008) (Fig. 4a). The results of subgroup analysis showed statistical differences in pelvic surgery (WMD = 0.671, 95% CI: 0.103 to 1.240, *P* = 0.021) and high-quality (WMD = 0.576, 95% CI: 13.594 to 39.558, *P* < 0.001) (Fig. 4b and c).

### Duration of postoperative analgesia

**Fig. 5:**
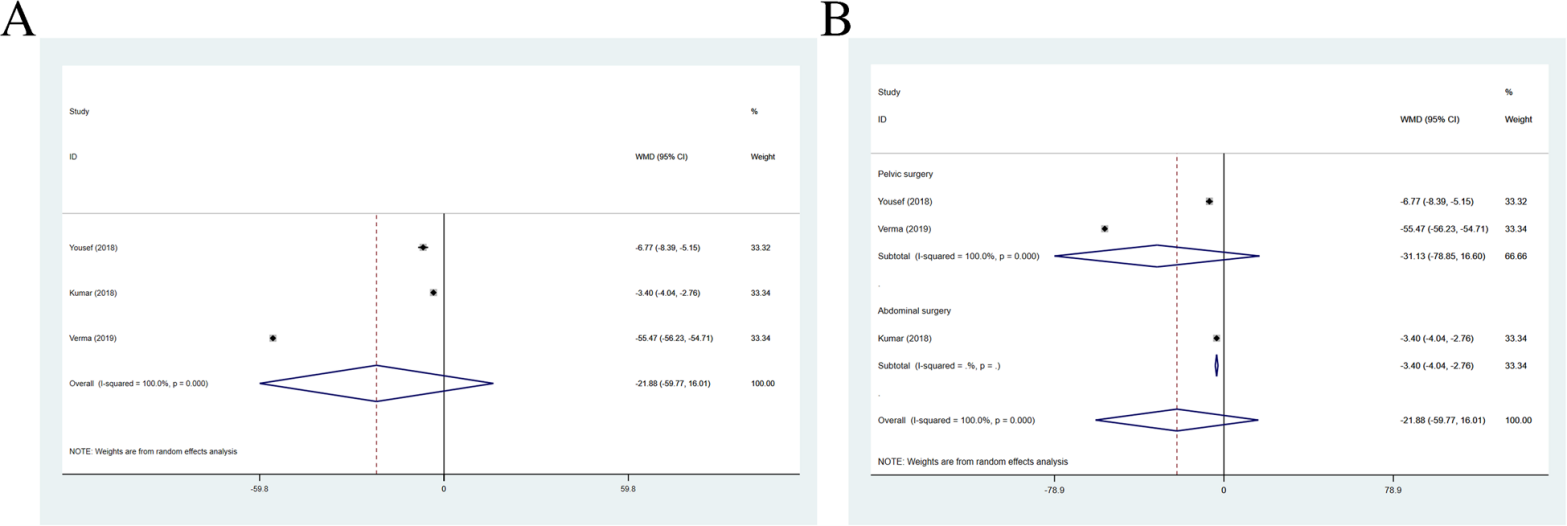
Forest plot for duration of postoperative analgesia (**a**) and operation types (**b**)

The duration of postoperative analgesia (h) was reported as an outcome in 3 studies (*n* = 190) (I^2^ = 100.00%). Duration of postoperative analgesia in TAPB group was shorter than QLB group (WMD = -21.882, 95% CI: − 59.774 to 16.010, *P* = 0.258) (Fig. 5a). The findings also showed differences in abdominal surgery (WMD = -3.400, 95% CI: − 4.038 to − 2.762, *P* < 0.001) (Fig. 5b).

### The number of patients requiring analgesia postoperatively

Nine studies (564 patients) on the number of patients requiring analgesia postoperatively were analyzed (I^2^ = 13.2%). The results founded that the number of patients requiring analgesia after surgery in TAPB group were higher than QLB group (RR = 2.618, 95% CI: 2.040 to 3.361, *P* < 0.001).

### Adverse reactions

**Fig. 6:**
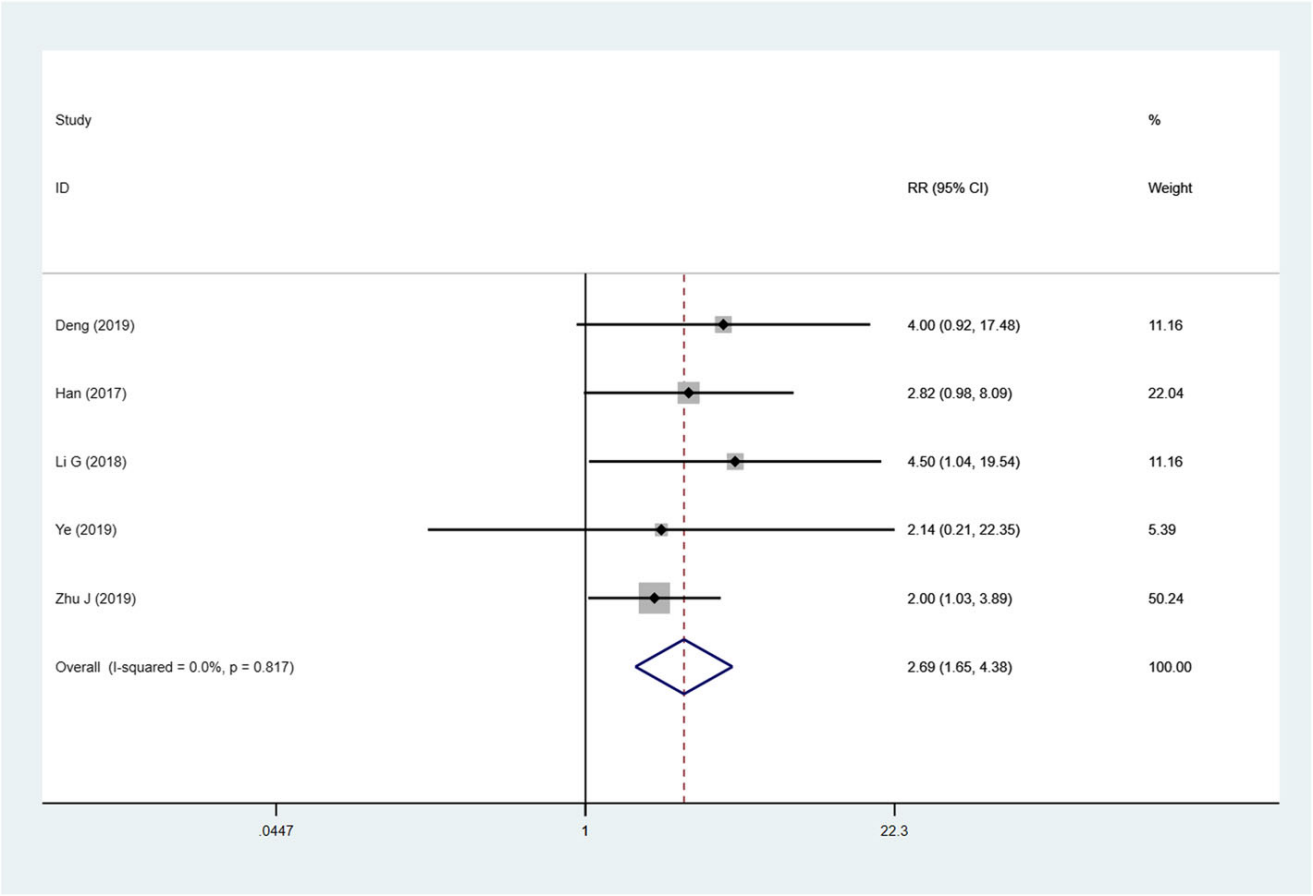
Forest plot for the incidence of dizziness

The incidence of dizziness in TAPB group from 5 articles was (*n* = 361) higher than that in QLB group (I^2^ = 0.0%, RR = 2.691, 95% CI: 1.653 to 4.382, *P* < 0.001) (Fig. 6). 8 studies (*n* = 535) on the incidence of nausea and vomiting were no differences between the two groups (I^2^ = 50.9%, RR = 1.918, 95% CI: 0.805 to 4.571, *P* = 0.141).

### Publication bias

Publication bias was performed using Begg’ test. There were no distinct publication bias in morphine consumption (Z = 1.36, *P* = 0.174), operative time (Z = 1.17, *P* = 0.240), duration of anesthesia (Z = 1.00, *P* = 0.317), VAS score at 24 h postoperatively (Z = 1.10, *P* = 0.273), duration of postoperative analgesia (Z = -1.00, *P* = 0.317), the number of patients requiring analgesia postoperatively (Z = -0.42, *P* = 0.677), the incidence of dizziness (Z = 0.49, *P* = 0.624), and nausea and vomiting (Z = -0.12, *P* = 1.000), except fentanyl consumption (Z = 2.23, *P* = 0.026).

## Discussion

Twenty-two studies [6, 15–20] on effects of TAPB vs. QLB in postoperative analgesia were included in this meta-analysis. Overall results showed that QLB showed more effective analgesia than TAPB in regards to morphine consumption, fentanyl consumption, VAS score at 24 h postoperatively, the number of patients requiring analgesia postoperatively, and the incidence of dizziness.

Pain was regarded as the fifth vital sign by the joint commission on accreditation of medical institutions (JCAHO) in 2000, ignoring pain management equals disrespecting human rights [36]. Postoperative pain is a major concern for patients and clinicians. Inadequate management of postoperative pain remains a common clinical problem worldwide [3, 4, 37]. TAPB has been described as a successful adjunct procedure for postoperative analgesia, however with some complications: failure of block, abdominal organ injury, nerve injury, vascular injury and so on [38–40]. Fortunately, the application of ultrasound can display injection point, the tap plane and the needle. With the guidance of ultrasound, the accuracy of puncture is improved, and the related complications are reduced [13, 41]. However, TAPB only blocks the anterolateral skin, muscles and parietal peritoneal sensory nerve fibers of the abdominal wall, and has no inhibitory effect on visceral pain [42]. QLB, as an effective and reliable option for relieving postoperative pain, is performed exclusively under the guidance of ultrasound, and the passage of the needle and the site of the local anesthetic application are far from the abdominal organs, great vessels and peritoneal cavity [43–47]. QLB can block the sympathetic nerves distributed between the thoracolumbar fascias. Some scholars suggested that QLB may alleviate the visceral pain to a certain extent [48]. Compared with TAPB, the drug diffusion range of QLB drugs was relatively wide, even reaching the paravertebral space of chests [49].

In this meta-analysis, VAS score at 24 h postoperatively of TAPB group was higher than that of QLB group, which may cause high consumption of analgesics. We also found that morphine and fentanyl consumption postoperatively in TAPB group were higher than QLB group. Similarly, a previous study showed that QLB type 1 significantly reduced morphine consumption up to postoperative 48 h [50]. Salama et al. found that QLB performed after cesarean section provided an ideal effect in reducing total postoperative morphine consumption [44]. The reason why the patients in TAPB group consumed more morphine than QLB group may be that TAPB only provides effective somatic analgesia, however poor effect in visceral analgesia [47]. Yousef reported that patients undergoing hysterectomy bilateral QLB provided more effective intraoperative and postoperative analgesia with less intraoperative fentanyl consumption and less postoperative morphine consumption compared with bilateral TAPB [18]. As we all know, morphine and fentanyl are common analgesic drugs for pain, and excessive use may cause several adverse reactions. Herein, it is significant for postoperative analgesia to explore an adjunct procedure that can reduce analgesia consumption.

The number of patients requiring analgesia postoperatively in QLB group was less than TAPB group. Zhu et al. [51] have studied the rate at patients who receive QLB requested analgesia postoperatively. They performed ultrasound-guided subcostal approach to QLB in an ipsilateral parasagittal oblique plane at the L1-L2 level on patients who underwent laparoscopic nephrectomy, and they reported that QLB was related with reducing rate of patients requiring rescue analgesia postoperatively. There were no significant differences in the operative time, duration of anesthesia, duration of postoperative analgesia, and nausea and vomiting between the two groups. The reasons may be less number of articles and small sample size included in this study. More high-quality studies with large samples are needed to further verify these results.

Because of representing the high level of evidences, the meta-analysis of RCTs can help patients, doctors and policy-makers to make decisions [52]. This meta-analysis was conducted to compare the effect of TAPB and QLB on postoperative analgesia based on RCT studies. However, several limitations of this study should be noted. First, heterogeneity existed in some measurements, and subgroup analyses failed to change the heterogeneity. Furthermore, there was a publication bias in fentanyl consumption, which may be attributed to the fact that the positive results were easy to publish, and only one English article and 2 low-quality studies were included in this meta-analysis. These factors mentioned above may affect our results. Therefore, the current results should be interpreted with caution.

## Conclusions

In summary, compared with TAPB, QLB provided effective intraoperative and postoperative analgesia with less morphine consumption, less fentanyl consumption, lower VAS score at 24 h postoperatively, decreased number of patients requiring analgesia postoperatively, and reduced incidence of dizziness. In addition, QLB is comparable with TAPB as regards to operative time, duration of anesthesia, and the incidence of nausea and vomiting. More researches with well-designed and adequate sample size are required to confirm these findings.

## Data Availability

All data generated or analyzed during this study are included in this published article.

## Abbreviations

TAPB: Transversus abdominis plane block
QLB: Quadratus lumborum block
RCTs: Randomized controlled trials
VAS: Visual analogue scale
RR: Relative risk
WMD: Weighted mean difference
JCAHO: Joint commission on accreditation of medical institutions
