# Correction to: Effects of transversus abdominis plane block versus quadratus lumborum block on postoperative analgesia: a meta-analysis of randomized controlled trials

**DOI:** 10.1186/s12871-020-01047-1

**Published:** 2020-05-27

**Authors:** Yanqing Wang, Xiaojia Wang, Kexian Zhang

**Affiliations:** 1 Department of Anesthesiology, Sichuan Cancer Hospital & Institute, SichuanCancer Center, School of Medicine, University of Electronic Science andTechnology of China, No.55, Section 4, South Renmin Road, Chengdu, 610041 People’s Republic of China; 2 Department of Pain management, WestChina Hospital, Sichuan University, Chengdu, 610041 People’s Republic of China

**Correction to: BMC Anesthesiology 20, 103 (2020)**

**https://doi.org/10.1186/s12871-020-01000-2**

Following publication of the research article [1], we have been notified that some values were given incorrectly. In the section “Abstract-Results” stated that: the number of patients requiring analgesia postoperatively (WMD = 3.893, 95%CI: 2.053 to 5.733, *P* < 0.001), this values are incorrect and should be expressed as follows: WMD = 2.618, 95%CI: 2.040 to 3.361, *P* < 0.001. In the section “Results-Overall meta-analysis” stated that: the number of patients requiring analgesia postoperatively (WMD = 3.893, 95%CI: 2.053 to 5.733, *P* < 0.001), this values are incorrect and should be expressed as follows: WMD = 2.618, 95%CI: 2.040 to 3.361, *P* < 0.001. In the section “Results-VAS score at 24 h postoperatively” stated that: high-quality (WMD = 0.576, 95%CI: 13.594 to 39.558, *P* < 0.001), this values are incorrect and should be expressed as follows: WMD = 0.576, 95%CI: 0.048 to 1.104, *P* = 0.032.

## References

[1] Wang Y (2020). Effects of transversus abdominis plane block versus quadratus lumborum block on postoperative analgesia: a meta-analysis of randomized controlled trials. BMC Anesthesiol.

